# Optimism and pessimism are antithetically associated with post-operative knee function in patients’ undergoing total knee arthroplasty

**DOI:** 10.1007/s00167-023-07434-8

**Published:** 2023-05-05

**Authors:** Felix Wunderlich, Jasmin Ghaemi Kerahrodi, Robert Kuchen, Thomas Klonschinski, Yama Afghanyar, Erik Wegner, Philipp Drees, Lukas Eckhard

**Affiliations:** 1 Department of Orthopedics and Traumatology, University Medical Center of the Johannes Gutenberg University Mainz, Langenbeckstrasse 1, 55131 Mainz, Germany; 2 Department of Psychosomatic Medicine and Psychotherapy, University Medical Center of the Johannes Gutenberg University Mainz, Mainz, Germany; 3 Institute of Medical Biostatistics, Epidemiology and Informatics, University Medical Center of the Johannes Gutenberg University Mainz, Mainz, Germany

**Keywords:** Optimism, Pessimism, Knee arthroplasty, Functional outcome, LOT-R, KOOS-12

## Abstract

**Purpose:**

Personality traits, such as dispositional optimism and pessimism, have impact on a variety of health-related problems. Influence on outcome in total knee arthroplasty (TKA) could only be shown for other personality trait concepts, but not for dispositional optimism/pessimism. This study aims to examine the association of dispositional optimism/pessimism with pre-operative joint function and post-operative outcome in TKA.

**Methods:**

Data were acquired in a multicentre, cross-sectoral, prospective study (the PROMISE Trial). Patients were followed for 12 months post-operatively. Dispositional optimism/pessimism was measured pre-operatively via the revised Life Orientation Test (LOT-R), pre- and post-operative function was measured via the 12 Item Knee-osteoarthritis outcome Scores (KOOS-12). Log-linear regression models considering known confounders and t-test were carried out to show the association of LOT-R scores with pre- and post-operative KOOS-12 scores.

**Results:**

740 patients were analyzed. Optimistic LOT-R was significantly positively associated to the mean scores of KOOS-12 pre- and post-operative, while pessimistic LOT-R was significantly associated negatively (pre-operative: optimistic *p* = 0.001, pessimistic *p* = 0.001; post-operative optimistic: 3M *p* = 0.001, 6M *p* = 0.001, 12M *p* = 0.001; post-operative pessimistic: 3M *p* = 0.01, 6M *p* = 0.004, 12M *p* = 0.001).

**Conclusion:**

Optimism was positively associated with pre-operative joint function and, more importantly, post-operative functional outcome in TKA, while pessimism was associated with the opposite. Assessing patients’ general personality traits prior to surgery to identify pessimistic patients, hence being at risk for poor outcome in TKA, should be considered to react to the patients’ special needs and possible pessimistic expectations, i.e., through a cognitive–behavioral intervention, to potentially increase optimism and hereby post-operative outcome in TKA.

**Level of evidence:**

Prognostic Level III.

## Introduction

While total knee replacement is an overall successful procedure for pain relief and improvement of mobility in patients suffering from end stage osteoarthritis (OA), literature still reports up to 30% of patients not being satisfied with their post-operative outcome [1, 2]. The causes for post-operative dissatisfaction can be diverse and are a central point of current research, as surgeons constantly seek to improve their patients satisfaction and function in total knee arthroplasty (TKA) [3, 4]. While more obvious causes, such as mechanical malalignment or implant loosening, are well studied and can be easily understood and remedied, there are still patients being unsatisfied without any apparent reason [5]. Former research stated that patients’ psychosocial factors have an influence on post-operative outcome [6–8], but data is scarce, often biased, and inconsistent, mostly because of the difficult separability of psychosocial factors and their mutual confounding.

Other research has shown that certain personality constructs have positive or negative impact on the outcome in a variety of health problems and the overall mortality [9–11], but did not investigate dispositional optimism/pessimism. The psychological constructs of dispositional optimism/pessimism are understood as a situation-related psychological constructs, with generalized inclination to expect favorable or unfavorable outcomes in life, meaning that a positive expectancy results in a higher patient effort for reaching desired goals, whereas negative expectancy determines the opposite [12]. Balck et al. [13] showed positive influence of dispositional optimism on post-operative function in total hip arthroplasty. While other personality constructs and their influence on joint replacement have been studied extensively [4, 14, 15], no studies investigating the influence of optimism and pessimism on TKA exist to date. To understand how the predetermined personality constructs of dispositional optimism/pessimism affect the overall satisfaction after TKA, this study aims to assess if optimism and pessimism affect the patients’ self-reported pre- and post-operative joint function in TKA. It was hypothesized that optimistic patients would have better post-operative function than their pessimistic counterparts throughout the first post-operative year.

## Materials and methods

All study data were derived from a multicenter, cross-sectoral, prospective healthcare research study (the PROMISE Trial) which was implemented in three different German hospitals, representing all levels of care. Sample size was chosen as available. Detailed methods descriptions (e.g., surgical techniques, rehabilitation procedures, patient education, etc.) are available via the published PROMISE Trial study protocol [16].

### Criteria for inclusion and exclusion

After Institutional Review Board approval, all patients older than 18 years that met standardized criteria for surgery [17] and were scheduled for primary TKA due to end stage OA were eligible for inclusion. After informed consent, they were enrolled in the study. Exclusion criteria were German language inability, noncompliance of signing a written consent, life expectancy less than 1 year as judged by the treating physician, and any health factors that would preclude elective surgery (i.e., decompensated cardiac insufficiency, symptomatic anemia, etc.). Patient withdrawal was accepted at any time during the trial.

### Data collection

**Fig. 1:**
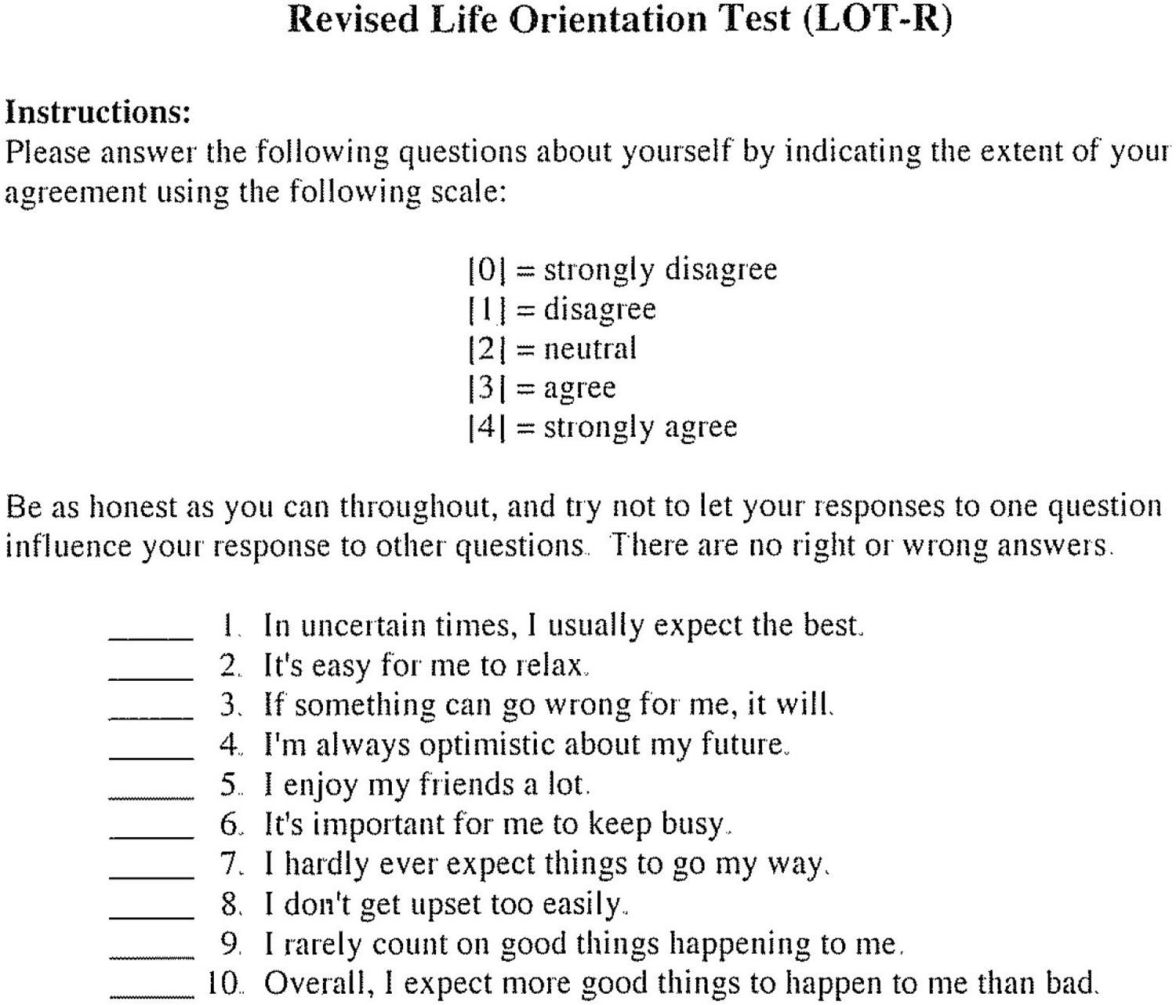
Revised Life Orientation Test (LOT-R)

Patients were followed for 12 months post-operatively during the regular administrative procedures of the participating hospitals. Evaluation started in 05/2018 and ended in 01/2021. Baseline data (age, age at surgery, sex, type of surgery, ASA score, BMI, marital status, education level and income) for the eligible patients were elicited before surgery. Patients’ personality dimensions were queried pre-operatively using the German version of the revised Life Orientation Test (LOT-R) (Fig. 1) [18]. The LOT-R is a widely used instrument to measure dispositional optimism and pessimism according to the model of Scheier and Carver published in 1985 [12]. The score consists of 10 items, with three items (1, 4 and 10) assessing optimism, three items (3, 7 and 9) assessing pessimism and four filler items. Answers are given on a 5-point Likert scale, response categories range from strongly agree to strongly disagree. The scores of the optimism and pessimism sub-scales are the sum of the scores of the corresponding items. A total score can be calculated, adding the optimism and the inverted pessimism score [19]. Its reliability and validity have been established [20] and norm values have been determined [21], while it measures concepts with a high test–retest reliability [22]. In this study, in case of the regression analysis, the LOT-R Score variable was used as a separate, continuously bi-dimensional variable for both pessimism and optimism, which is the preferred application of this test [20, 21]. High score levels on the separate scales of the both variables “optimism” and “pessimism” indicated “high optimism” or “low pessimism”, respectively. In case of the descriptive statistics, LOT-R was categorized. “Optimistic” was defined as the following: 0–6 points = not optimistic, 7–9 points = somewhat optimistic, 10–12 points = very optimistic, whereas “pessimistic” was defined as the following: 0–6 points = very pessimistic, 7–9 points = somewhat pessimistic, 10–12 points = not pessimistic. Functional outcome was measured via the 12-Item Knee Osteoarthritis Outcome Score (KOOS-12) pre-operative and at 3-, 6- and 12-months post-operative. The KOOS-12 score evaluates symptoms and function using items from three subscales: “pain”, “function”, daily living”, and “quality of life”. Items are scored on a 4-point Likert scale and scores range from 0 to 100, with higher scores indicating better results. The score and its German version have been validated and checked for reliability, and the minimum clinically important difference (MCID) was detected at 11.1 points [23–25].

The surveys (LOT-R, KOOS-12) were handed to included patients after informed consent during the last visit at the outpatient clinic prior to surgery (range 1–8 weeks), and post-operative at the above-described follow-ups. Patients completed the surveys on their own, with qualified study nurses on-site on demand if questions arose. Collected data was pseudonymized and stored electronically.

### Patient demographics

The data of 933 TKA patients were included in the study. At last follow-up, 740 patients with complete data could be analyzed. Baseline characteristics are shown in Table 1.

**Table 1:** Baseline characteristics

Variable	
Age (years) (mean [SD])	66.67 [10.11]
Age at surgery (years) (mean [SD])	67.1 [9.45]
Sex (*n*)	
Female	53.5% (396)
Male	46.5% (344)
BMI (kg/m^2^) (mean[SD])	30.24 [5.79]
ASA score (*n*)	
I	7.4% (54)
II	62.5% (458)
III	29.5% (216)
IV	0.7% (5)
Missing	0.9% (7)
Marital status (*n*)	
Married	77.9% (576)
Not married	22.1% (163)
Missing	0.1% (1)
Education level (European baccalaureate) (*n*)	
Yes	14.4% (106)
No	85.6% (631)
Missing	0.4% (3)
Income	
< 2000€	26.4% (195)
> 2000€	50.1% (371)
Missing	23.5% (174)
LOS (days) (mean [SD])	5.34 [3.88]

*BMI* body mass index, *ASA* American Society of Anesthesiologists, *LOS* length of stay

### Institutional Review Board approval

Approval from all ethics committees in the participating states in Germany was obtained prior to the study [Institutional Review Board approval: Submission No.: 837.533.17 (11367), Ethics Committee at the State Chambers of Physicians of Rhineland-Palatinate; B-F-2018-042, Ethics Committee at the State Chambers of Physicians of Baden-Wuerttemberg; MC 84/2018, Ethics Committee at the State Chambers of Physicians of Hesse].

### Statistical analysis

To quantify the possible impact of LOT-R optimism/pessimism on post-operative outcomes, several linear regression models were fitted, in which the respective outcomes (KOOS-12 post-operative) were regressed on the study variable LOT-R optimism/pessimism. In addition to the respective study variable, each regression model included the possible confounders: age, ASA score, BMI, pre-operative KOOS-12 at baseline, EQ5D score, HSS expectation score, education background (low vs. high) and sex as covariates.

To obtain the regression equation for the association of LOT-R pessimism with the respective KOOS-12 score, the LOT-R- optimism score was replaced with the LOT-R-pessimism score of a given patient.

To prevent an attrition bias, all regression models were based on 10 datasets that were obtained by means of multiple imputation (using predictive mean matching as the imputation method). The imputation model included all recorded variables that were significantly correlated with LOT-R optimism/pessimism and the KOOS-variable.

All statistical analyses were performed using R [R version 3.5.1, Core Team (2017)] [26]. The level of significance was set at 0.05. While continuous variables were summarized with their mean and standard deviation, categorical variables were summarized with numbers and percentages.

## Results

### Personality traits

**Fig. 2:**
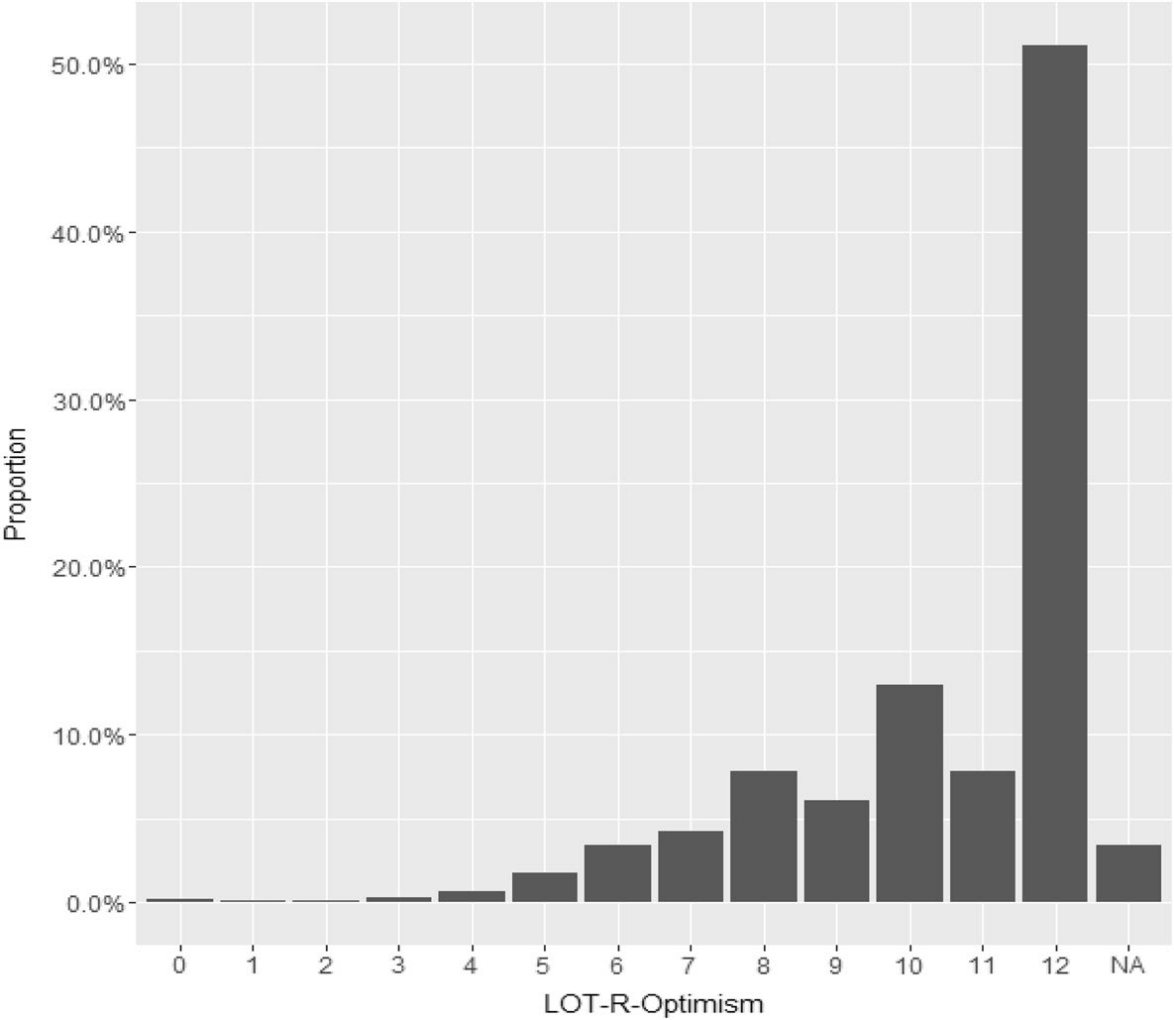
Total distribution of LOT-R optimism in the cohort. Point value > 10 indicates “very optimistic”, 7–9 indicates “somewhat optimistic”, and < 7 “not optimistic”

**Fig. 3:**
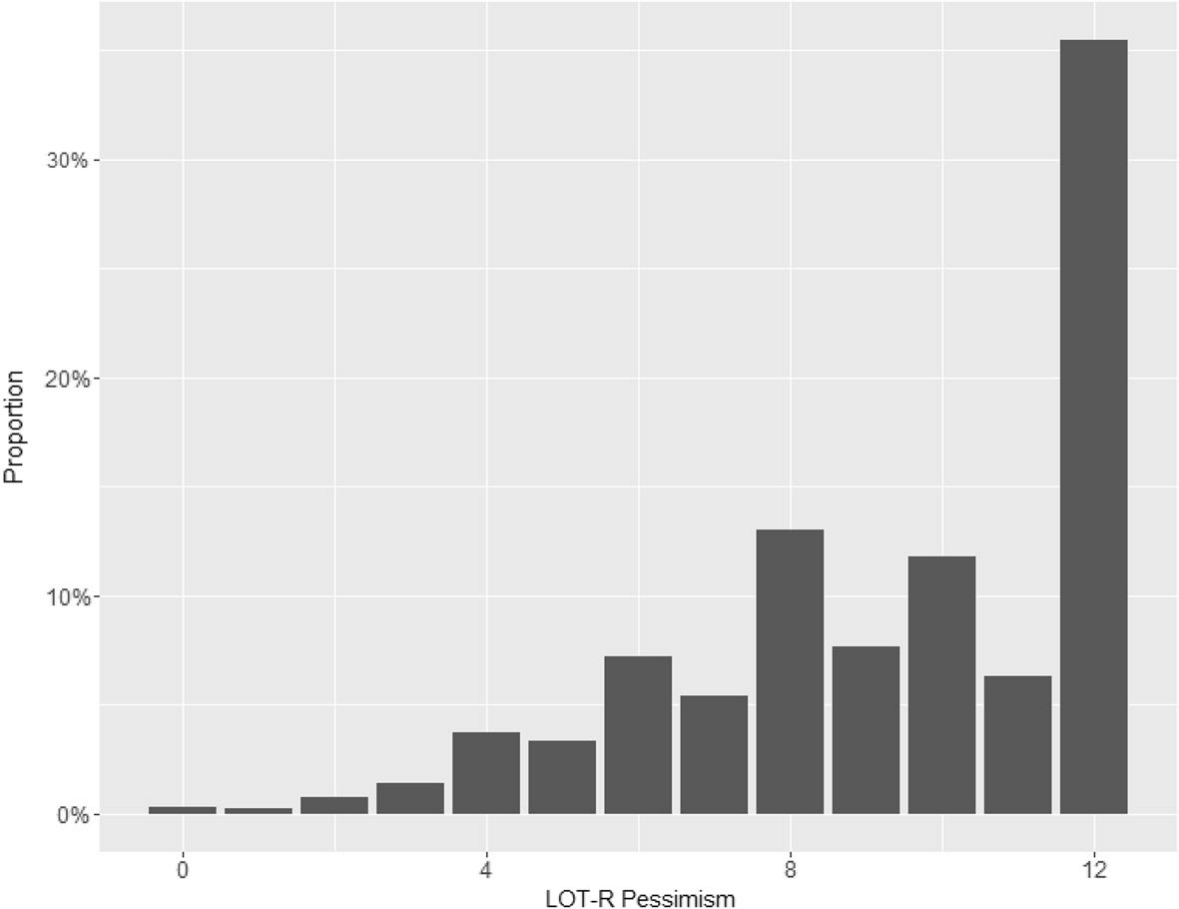
Total distribution of LOT-R pessimism in the cohort. Point value > 10 indicates “not pessimistic”, 7–9 “rather pessimistic”, and < 7 “pessimistic”

Mean LOT-R optimism was 10.5 (SD 2.12). Mean LOT-R pessimism was 9.4 (SD 2.03). Distributions of LOT-R optimism and LOT-R pessimism are shown in Figs. 2 and 3.

### Mean functional scores

Mean KOOS Scores for pre- and post-operative follow-ups in association with the respective optimistic and pessimistic categories are shown in Tables 2 and 3.

**Table 2:** Mean KOOS-12 scores in LOT-R optimistic values

	Very optimistic (*N* = 436)	Somewhat optimistic (*N* = 210)	Not optimistic (*N* = 94)	Total (*N* = 740)
KOOS pre-OP				
N-Miss	4	3	0	7
Mean (SD)	39.11 (13.55)	35.66 (13.70)	30.90 (14.76)	37.08 (14.02)
KOOS 3 months post OP				
N-Miss	77	32	19	128
Mean (SD)	66.99 (17.44)	59.64 (18.14)	53.45 (16.38)	63.19 (18.16)
KOOS 6 months post OP				
N-Miss	82	37	21	140
Mean (SD)	72.61 (18.75)	65.40 (19.06)	63.53 (17.72)	69.43 (19.08)
KOOS-12 months post OP				
N-Miss	120	61	33	214
Mean (SD)	78.91 (17.97)	72.65 (18.32)	68.23 (21.70)	75.90 (18.91)

**Table 3:** Mean KOOS-12 scores in LOT-R pessimistic values

	Not pessimistic (*N* = 308)	Somewhat pessimistic (*N* = 245)	Very pessimistic (*N* = 184)	Total (*N* = 737)
KOOS pre-OP				
N-Miss	0	2	4	6
Mean (SD)	40.56 (13.76)	36.75 (12.93)	31.89 (14.13)	37.16 (13.99)
KOOS 3 months post OP				
N-Miss	44	45	37	126
Mean (SD)	65.96 (16.28)	62.79 (18.03)	59.36 (20.72)	63.34 (18.17)
KOOS 6 months post OP				
N-Miss	46	48	43	137
Mean (SD)	72.69 (17.43)	68.36 (18.97)	65.40 (21.01)	69.56 (19.03)
KOOS-12 months post OP				
N-Miss	66	76	69	211
Mean (SD)	79.44 (16.58)	74.98 (18.08)	70.29 (22.62)	76.01 (18.84)

### Pre-operative function and association with dispositional optimism/pessimism

**Fig. 4:**
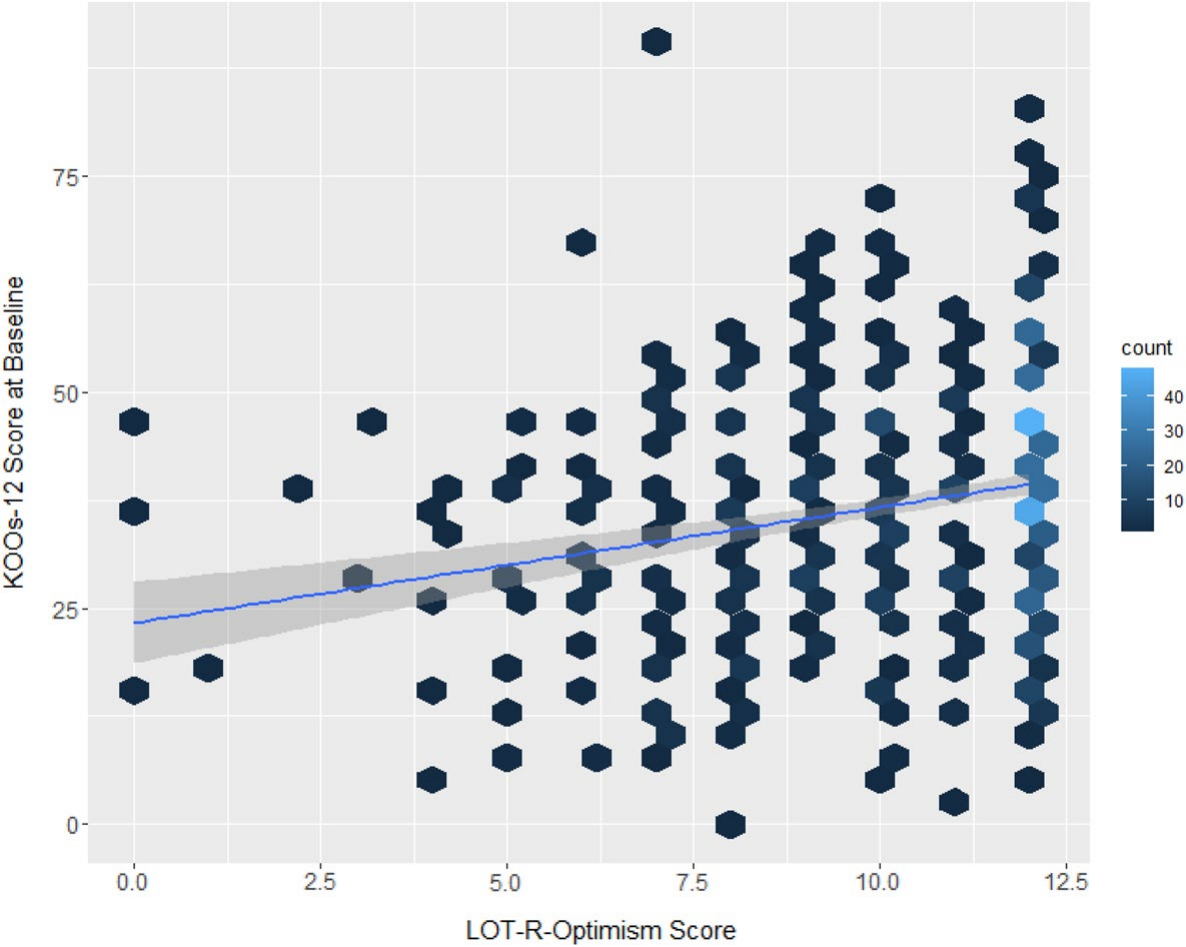
Association between the LOT-R-optimism values and the pre-operative KOOS-12 values in the cohort. Hexagons indicate patient clusters with lighter colors indicating a higher number of patients. The continuous line indicates the slope of a univariate regression model, while the surrounding, transparent area corresponds to the standard error of the slope coefficient

**Fig. 5:**
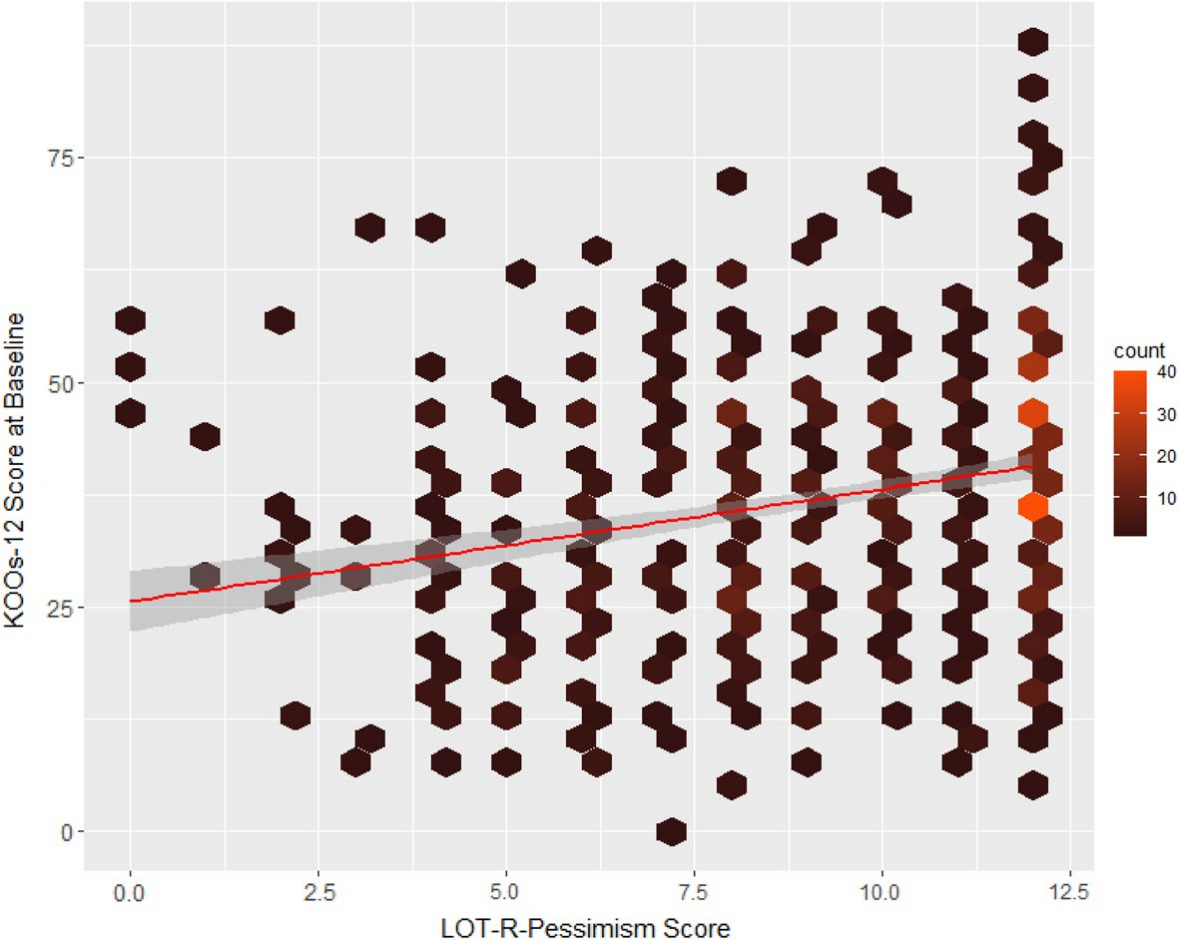
Association between the LOT-R-pessimism values and the pre-operative KOOS-12 values in the cohort. Hexagons indicate patient clusters with lighter colors indicating a higher number of patients. The continuous line indicates the slope of a univariate regression model, while the surrounding, transparent area corresponds to the standard error of the slope coefficient

Optimistic patients showed significantly higher pre-operative KOOS-12 scores compared to their not optimistic counterparts (*p* ≤ 0.001) (Fig. 4). Pessimistic patients showed significantly lower pre-operative KOOS-12 scores compared to their not pessimistic counterparts (*p* ≤ 0.001) (Fig. 5).

### Post-operative function and association with dispositional optimism/pessimism

**Fig. 6:**
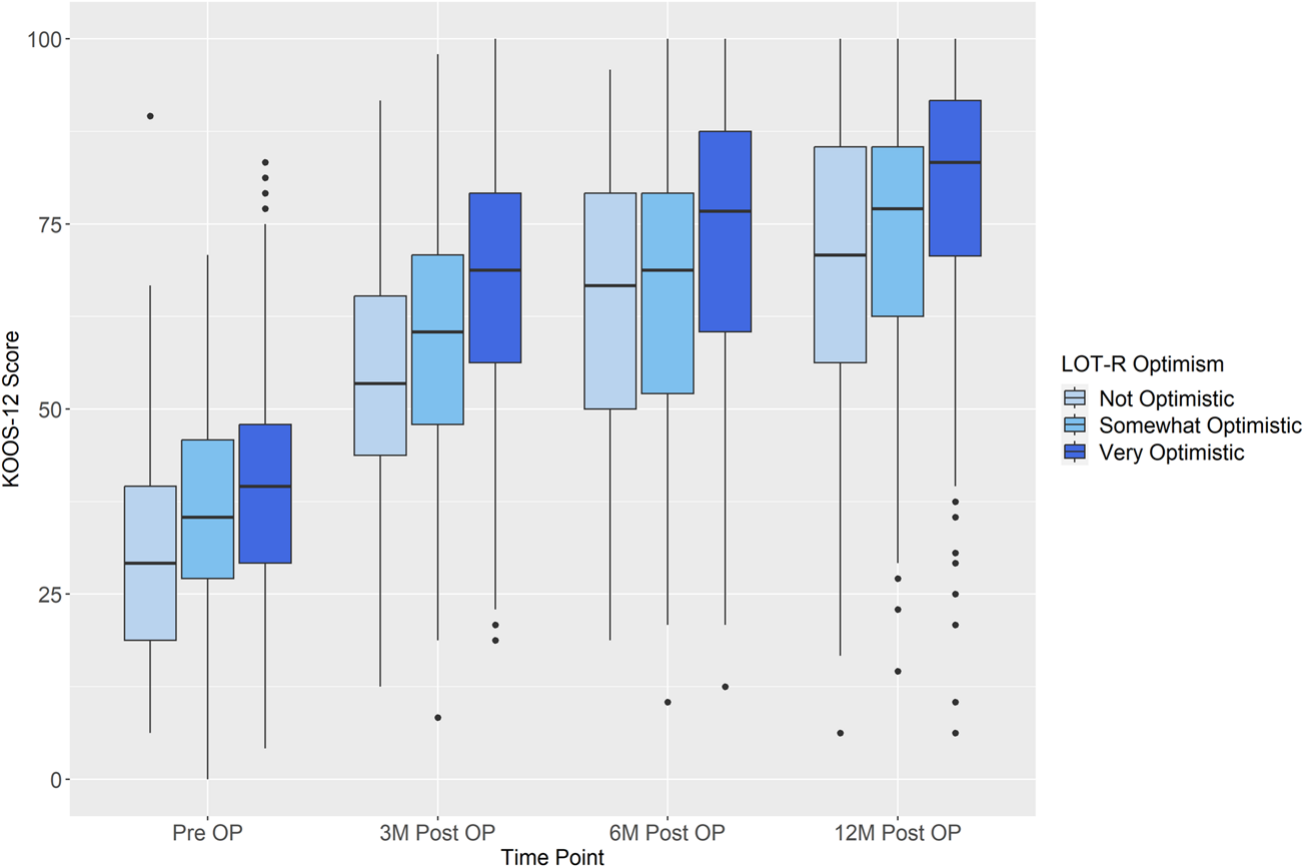
Association of LOT-R optimism and pre- and post-operative absolute KOOS-12 values

**Fig. 7:**
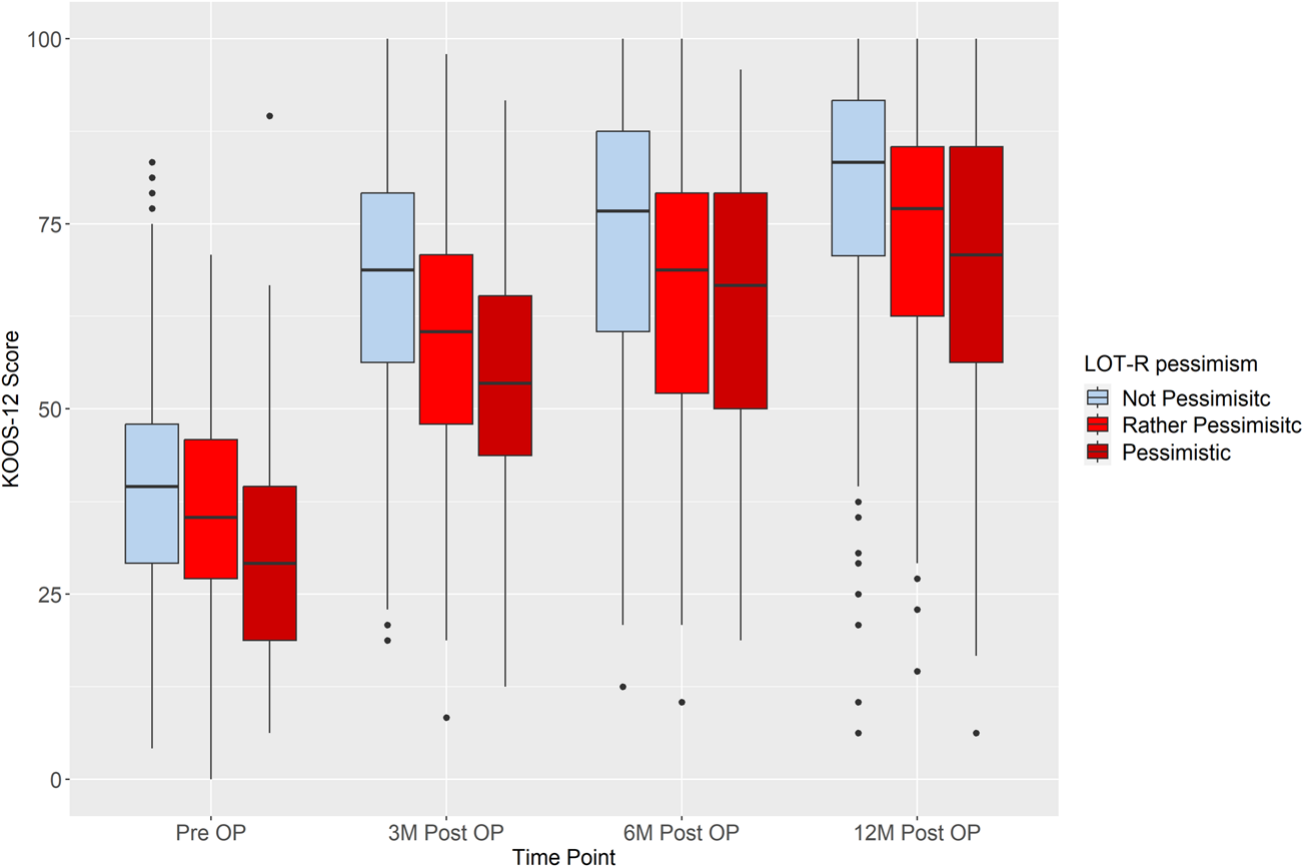
Association of LOT-R pessimism and pre- and post-operative absolute KOOS-12 values

Associations of LOT-R optimism and LOT-R pessimism scores with absolute post-operative KOOS-12 scores (3, 6 and 12 months) in the multivariate analysis are shown in Table 4, Figs. 6 and 7). Further analysis of the association of post-operative function and optimism/pessimism in the univariate analysis is shown in Table 5. This analysis showed an overall low positive correlation of LOT-R optimism and LOT-R pessimism with KOOS-12 values, respectively (*R*^2^-values shown in Tables 4 and 5).

**Table 4:** Association of optimism and pessimism with 3, 6 and 12 months post-operative absolute KOOS-12 scores, when adjusting for the confounders (i.e., KOOS-12 baseline value)

	LOT-R optimistic (*N* = 436)			LOT-R pessimistic (*N* = 429)		
	*R*^2^	*β* value	*p* value	*R*^2^	*β* value	*p* value
Pre-op KOOS-12	0.50	0.65	< 0.001**	0.5	0.54	< 0.001**
KOOS-12 3 M post-op	0.13	1.55	< 0.001**	0.10	0.65	0.012**
KOOS-12 6 M post-op	0.10	1.23	< 0.001**	0.10	0.83	0.004**
KOOS-12 12 M post-op	0.09	1.14	< 0.001**	0.10	1.09	< 0.001**

**Indicates statistical significance

**Table 5:** Association of optimism and pessimism with 3, 6 and 12 months post-operative absolute KOOS-12 scores without adjusting for confounders

	LOT-R optimistic (*N* = 436)			LOT-R pessimistic (*N* = 429)		
	*R*^2^	*β* value	*p* value	*R*^2^	*β* value	*p* value
Pre-op KOOS-12	0.046	1.34	< 0.001**	0.064	1.25	< 0.001**
KOOS-12 3M post-op	0.063	2.03	< 0.001**	0.025	1.01	< 0.001**
KOOS-12 6M post-op	0.044	1.78	< 0.001**	0.033	1.23	< 0.001**
KOOS-12 12M post-op	0.029	1.44	< 0.001**	0.038	1.31	< 0.001**

**Indicates statistical significance

## Discussion

The most important findings of this study were the significant positive/negative association of dispositional optimism/pessimism with post-operative functional outcome after TKA measured via the KOOS-12 score. Optimistic patients showed significant higher KOOS-12 scores pre-operative as well as 3, 6 and 12 months post-operative, while pessimistic patients had significant lower scores at all survey points compared to their respective counterparts.

The findings of higher post-operative function in optimistic than in pessimistic patients are in line with other literature, showing better outcome in optimistic patients after total hip arthroplasty [13] and faster recovery of optimistic patients from coronal bypass surgery [27]. Other studies associated optimism with lower pain scores and an overall higher quality of life [28]. Supportively, Singh et al. identified pessimism as a risk factor for poor pain and functional outcome after joint replacement [29], whereas Novotny et al. showed poorer survival in pessimistic patients suffering from lung cancer [10]. These facts support the above-mentioned findings, as the KOOS-12 score uses items assessing joint pain and overall quality of life.

Nevertheless, the significant higher post-operative functional scores in optimistic patients could be derived by the influence of pre-operative functional score levels on post-operative score values as this has been shown previously [30]. This potential bias was eliminated by conducting analysis with pre-operative functional scores as potential confounder, which did not alter statistical significance.

Besides the overall higher satisfaction in life and after surgery in optimists [22], there might be several reasons for better functional outcome in optimists after TKA. Dispositional optimism is partly defined as expecting favorable outcomes for life events, implying higher expectations regarding the outcome of medical interventions, which Tolk et al. [4] were also able to prove in case of TKA. Tilbury et al. [31] showed that higher expectations on total joint replacement were associated with more favorable outcomes with less pain and better function after surgery. Furthermore, one could assume that optimistic patients show greater resilience for difficulties occurring after joint replacement and therefore develop better coping strategies. Carver et al. linked optimism with higher levels of engagement coping, since optimistic patients are more likely to set goals for recovery even before surgery [22]. Considering these findings, greater motivation for recovery might lead to more effort in rehabilitation programs and self-exercising, potentially determining better post-operative functional scores. Another potential reason could be the interaction of optimism and social support in a way that optimists appear more attractive to others than pessimists, and therefore receiving more social support and better treatment [32]. The above-mentioned considerations can be applied reciprocally when analyzing the influence of pessimism on functional outcome, as pessimism is known to predict less outcome expectations in total joint arthroplasty [4] and pessimistic patients, as known in patients suffering from depression [29], might not adhere sufficiently to physical rehabilitation.

Even though the KOOS-12 is a widely used instrument in patient-reported outcome measurements (PROMs), one has to take into consideration that PROMs might not be the right instrument when measuring outcome in optimists and pessimists. Scherer et al. [33] stated that optimistic patients focus less on the negative aspects of the experience, both distress and symptoms. If this applies to every optimistic patient, PROMs could be limited under these circumstances and could always show better results in optimistic than in pessimistic patients, although the objective assessment of joint function might be equal or even superior in pessimistic patients.

When considering the study results, the question about the practical significance for orthopedic surgeons remains, especially as the MCID of the KOOS-12 was not reached at every post-operative survey point in this study, probably due to the large sample size. Nevertheless, our findings suggest considering every patient’s individual personality traits when he or she is admitted to surgery. This would allow the treating physicians to detect pessimistic patients, who tend to have worse outcomes and potentially react to their special needs and pessimistic expectations prior to surgery. A redirection of these pessimistic patients to a cognitive–behavioral intervention or an intervention on pessimism-predicting variables (e.g., preexisting mental disorders, lack of social support) prior to surgery could help to reduce pessimism and increase optimism and optimistic expectations in those patients, and thus could improve post-operative outcome.

### Limitations

This study has some limitations. First, other psychological variables known to interfere with post-operative outcome, such as depression and anxiety [6, 34], could not be taken into account, as they were not individually recorded in this database. Second, due to the character of an observational study without intervention, only associations and no causations could be investigated. Third, only PROMs and no objective outcome measures were used for measurement of post-operative functional outcome. Fourth, potential selection bias exists, as only patients already planned to undergo TKA were examined in this study.

However, due to the multi-center design, a large study sample size matching the general population of TKA patients in Germany [35] could be analyzed. It is notable that the optimism/pessimism survey took place close to surgery date (maximum 8 weeks pre-operative), suggesting high correlation with the patients’ actual current personality trait, especially as optimism/pessimism is known as a stable trait with high test–retest reliability [22]. Furthermore, treatment pathway and follow-up treatment were standardized in all participating study centers.

## Conclusions

Dispositional optimism is positively associated with pre-operative joint function and, more importantly, post-operative functional outcome in TKA, whereas the opposite applies for pessimism. Assessing patients’ general personality traits prior to surgery to identify pessimistic patients, hence being at risk for poor outcome in TKA, should be considered to react to the patients’ special needs and possible pessimistic expectations, i.e., through a cognitive–behavioral intervention, to potentially increase optimism and hereby post-operative outcome in TKA.

## Data Availability

The data that support the findings of this study are available on request from the corresponding author, FW. The data are not publicly available due to the German privacy laws in the medical sector.

## Abbreviations

TKA: Total knee arthroplasty
LOT-R: Life Orientation Test-Revised
KOOS-12: Knee osteoarthritis outcome score 12 Item
OA: Osteoarthritis
BMI: Body mass index
ASA: American Society of Anesthesiologists
MCID: Minimum clinically important difference
SD: Standard deviation
PROMs: Patient-reported outcome measurements

## References

[1] Bourne RB, Chesworth BM, Davis AM, Mahomed NN, Charron KDJ (2010). Patient satisfaction after total knee arthroplasty: who is satisfied and who is not?. Clin Orthop Relat Res.

[2] Conner-Spady BL, Bohm E, Loucks L, Dunbar MJ, Marshall DA, Noseworthy TW (2020). Patient expectations and satisfaction 6 and 12 months following total hip and knee replacement. Qual Life Res.

[3] Lützner C, Postler A, Beyer F, Kirschner S, Lützner J (2019). Fulfillment of expectations influence patient satisfaction 5 years after total knee arthroplasty. Knee Surg Sports Traumatol Arthrosc.

[4] Tolk JJ, Janssen RPA, Haanstra TM, Van Der Steen MMC, Bierma Zeinstra SMA, Reijman M (2020). Outcome expectations of total knee arthroplasty patients: the influence of demographic factors, pain, personality traits, physical and psychological status. J Knee Surg.

[5] Scott CEH, Howie CR, MacDonald D, Biant LC (2010). Predicting dissatisfaction following total knee replacement: a prospective study of 1217 patients. J Bone Jt Surg Br.

[6] Belford K, Gallagher N, Dempster M, Wolfenden M, Hill J, Blaney J (2020). Psychosocial predictors of outcomes up to one year following total knee arthroplasty. Knee.

[7] Bletterman AN, de Geest-Vrolijk ME, Vriezekolk JE, Nijhuis-van der Sanden MW, van Meeteren NLU, Hoogeboom TJ (2018). Preoperative psychosocial factors predicting patient’s functional recovery after total knee or total hip arthroplasty: a systematic review. Clin Rehabil.

[8] Vissers MM, Bussmann JB, Verhaar JAN, Busschbach JJV, Bierma-Zeinstra SMA, Reijman M (2012). Psychological factors affecting the outcome of total hip and knee arthroplasty: a systematic review. Semin Arthritis Rheum.

[9] Maruta T, Colligan RC, Malinchoc M, Offord KP (2000). Optimists vs pessimists: survival rate among medical patients over a 30-year period. Mayo Clin Proc.

[10] Novotny P, Colligan RC, Szydlo DW, Clark MM, Rausch S, Wampfler J (2010). A pessimistic explanatory style is prognostic for poor lung cancer survival. J Thorac Oncol.

[11] Petersen LR, Clark MM, Novotny P, Kung S, Sloan JA, Patten CA (2008). Relationship of optimism–pessimism and health-related quality of life in breast cancer survivors. J Psychosoc Oncol.

[12] Scheier MF, Carver CS (1985). Optimism, coping, and health: assessment and implications of generalized outcome expectancies. Health Psychol.

[13] Balck F, Lippmann M, Jeszenszky C, Günther KP, Kirschner S (2016). The influence of optimism on functionality after total hip replacement surgery. J Health Psychol.

[14] Haanstra TM, Tilbury C, Kamper SJ, Tordoir RL, Vlieland TPMV, Nelissen RGHH (2015). Can optimism, pessimism, hope, treatment credibility and treatment expectancy be distinguished in patients undergoing total hip and total knee arthroplasty?. PLoS One.

[15] Singh JA, O’Byrne MM, Colligan RC, Lewallen DG (2010). Pessimistic explanatory style: a psychological risk factor for poor pain and functional outcomes two years after knee replacement. J Bone Jt Surg Br.

[16] Betz U, Langanki L, Heid F, Spielberger J, Schollenberger L, Kronfeld K (2021). The PROMISE study protocol: a multicenter prospective study of process optimization with interdisciplinary and cross-sectoral care for German patients receiving hip and knee endoprostheses. Acta Orthop.

[17] Schmitt J, Lange T, Günther K-P, Kopkow C, Rataj E, Apfelbacher C (2017). Indication criteria for total knee arthroplasty in patients with osteoarthritis—a multi-perspective consensus study. Z Orthop Unfall.

[18] Scheier MF, Carver CS, Bridges MW (1994). Distinguishing optimism from neuroticism (and trait anxiety, self-mastery, and self-esteem): a reevaluation of the Life Orientation Test. J Pers Soc Psychol.

[19] Glaesmer H, Rief W, Martin A, Mewes R, Brähler E, Zenger M (2012). Psychometric properties and population-based norms of the Life Orientation Test Revised (LOT-R). Br J Health Psychol.

[20] Herzberg PY, Glaesmer H, Hoyer J (2006). Separating optimism and pessimism: a robust psychometric analysis of the revised Life Orientation Test (LOT-R). Psychol Assess.

[21] Glaesmer H, Hoyer J, Klotsche J, Herzberg PY (2008). The German version of the Life-Orientation-Test (LOT-R) for dispositional optimism and pessimism. Eur J Health Psychol.

[22] Carver CS, Scheier MF, Segerstrom SC (2010). Optimism. Clin Psychol Rev.

[23] Eckhard L, Munir S, Wood D, Talbot S, Brighton R, Walter B (2021). The KOOS-12 shortform shows no ceiling effect, good responsiveness and construct validity compared to standard outcome measures after total knee arthroplasty. Knee Surg Sports Traumatol Arthrosc.

[24] Eckhard L, Munir S, Wood D, Talbot S, Brighton R, Walter WL (2021). Minimal important change and minimum clinically important difference values of the KOOS-12 after total knee arthroplasty. Knee.

[25] Gandek B, Roos EM, Franklin PD, Ware JE (2019). Item selection for 12-item short forms of the Knee injury and Osteoarthritis Outcome Score (KOOS-12) and Hip disability and Osteoarthritis Outcome Score (HOOS-12). Osteoarthr Cartil.

[26] R Developtment Core Team (2018). R: A language and environment for statistical computing.

[27] Scheier MF, Matthews KA, Owens JF, Magovern GJ, Lefebvre RC, Abbott RA (1989). Dispositional optimism and recovery from coronary artery bypass surgery: the beneficial effects on physical and psychological well-being. J Pers Soc Psychol.

[28] Hanssen MM, Peters ML, Vlaeyen JWS, Meevissen YMC, Vancleef LMG (2013). Optimism lowers pain: evidence of the causal status and underlying mechanisms. Pain.

[29] Singh JA, Colligan RC, O’Byrne MM, Lewallen DG (2016). Do pessimists report worse outcomes after total hip arthroplasty?. BMC Musculoskelet Disord.

[30] Alattas SA, Smith T, Bhatti M, Wilson-Nunn D, Donell S (2017). Greater pre-operative anxiety, pain and poorer function predict a worse outcome of a total knee arthroplasty. Knee Surg Sports Traumatol Arthrosc.

[31] Tilbury C, Haanstra TM, Verdegaal SHM, Nelissen RGHH, De Vet HCW, Vliet Vlieland TPM (2018). Patients’ pre-operative general and specific outcome expectations predict postoperative pain and function after total knee and total hip arthroplasties. Scand J Pain.

[32] Bozo Ö, Gündoǧdu E, Büyükaşik-Çolak C (2009). The moderating role of different sources of perceived social support on the dispositional optimism-posttraumatic growth relationship in postoperative breast cancer patients. J Health Psychol.

[33] Scherer KR, Wranik T, Sangsue J, Tran V, Scherer U (2004). Emotions in everyday life: probability of occurrence, risk factors, appraisal and reaction patterns. Soc Sci Inf.

[34] Trinh JQ, Carender CN, An Q, Noiseux NO, Otero JE, Brown TS (2021). Resilience and depression influence clinical outcomes following primary total joint arthroplasty. J Arthroplast.

[35] Grimberg A, Jansson V, Lützner J, Melsheimer O, Morlock M, Steinbrück A (2020) Annual report of the German arthroplasty registry (EPRD). https://www.eprd.de/fileadmin/user_upload/Dateien/Publikationen/Berichte/Jahresbericht2020-Web_2020-12-11_F.pdf. Accessed 19 Nov 2021

